# Frailty: An overlooked red flag in sports surgery

**DOI:** 10.1002/jeo2.70497

**Published:** 2025-11-04

**Authors:** Yavuz Şahbat, Merve Güner, Safa Gürsoy

**Affiliations:** ^1^ Department of Orthopedics and Traumatology Istinye University, Bahcesehir Liv Hospital Istanbul Turkiye; ^2^ Department of Internal Medicine, Division of Geriatric Medicine, Faculty of Medicine Istinye University Istanbul Turkiye; ^3^ Department of Orthopaedics and Traumatology Faculty of Medicine Acibadem Mehmet Ali Aydinlar University Istanbul Turkey

**Keywords:** fatty atrophy, frailty, ligaments, rotator cuff, shoulder surgery, sport surgeon

## Abstract

Frailty can be defined as a decline in physiological and biological reserves that does not correlate directly with chronological age. While the general understanding of what geriatricians and orthopaedic surgeons should know about frailty has been increasingly discussed in recent years, the specific knowledge required by sports and shoulder surgeons remains underexplored. This paper aims to highlight what a sports and shoulder surgeon should know about frailty. With advances in healthcare technologies, both life expectancy and healthy life expectancy have significantly increased. A growing proportion of older adults are engaging in sports activities, and sports‐related injuries in this population are becoming more common. In this context, frailty scales can be utilised to assess preoperative patient condition, anticipate intraoperative challenges, and predict postoperative complications, morbidity, and mortality risk. These tools can guide sports surgeons in understanding a patient′s functional capacity and physiological resilience independently of chronological age.

**Abbreviations**
AGS: The American College of Surgeons and the American Geriatrics Society
ASA: American Society of Anesthesiologists
BMD: bone mineral density
CFS: The Clinical Frailty Scale
CGA: comprehensive geriatric assessment
NSAIDs: nonsteroidal anti‐inflammatory drugs
PROs: patient‐reported outcomes scores
TKA: total knee arthroplasty

## INTRODUCTION

The aim of this study is to inform orthopaedic sports and shoulder surgeons about the concept of frailty and to synthesise current evidence from the literature. As the global population ages, individuals increasingly seek ways to age dynamically and healthily. Orthopaedic and orthopaedic sports procedures are now frequently performed in this demographic. While the question of what we need to know about frailty has long occupied the minds of geriatricians [81], in recent years, orthopaedic surgeons [36, 45] have also begun to pay increasing attention to this concept.

**FIGURE 1:**
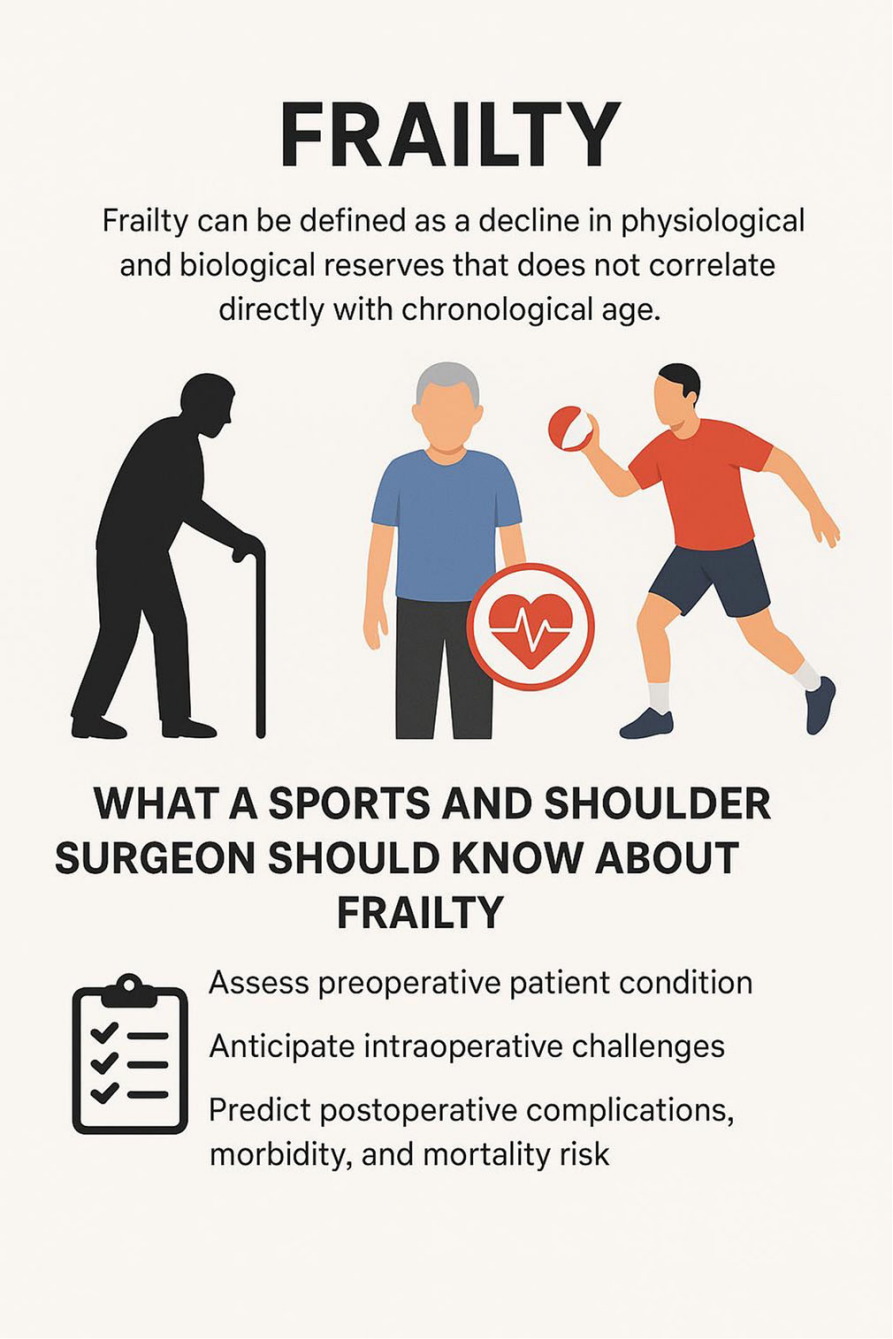
This infographic demonstrates the concept of frailty and its importance for sport and shoulder surgeons.

The concept of frailty has been extensively studied in trauma‐related procedures, particularly in hip fractures and arthroplasty patients, where higher frailty levels have been correlated with longer hospital stays, increased complication rates, and poorer clinical outcomes [36, 45, 73, 77]. However, sports‐related injuries are also becoming more common in the older population, and sports surgeons now increasingly encounter older adults with such injuries [36] (Figure 1).

In this technological era, the proportion of older patients undergoing surgery has risen in parallel with the demographic shift. As highlighted by Clegg et al., one in every five surgeries is performed on individuals aged 75 years and older [13]. Older adults face a higher risk of postoperative complications and mortality, emphasising the importance of accurate preoperative risk assessment. Frailty is prevalent not only in emergency settings but also in elective surgical contexts [23, 36]. With the increasing number and proportion of older adults requiring both elective and non‐elective surgical procedures, the concept and classification of frailty have emerged as important tools in orthopaedic and sports surgery to minimise unexpected postoperative outcomes.

Although chronological age is a well‐known risk factor, frailty is not an inevitable outcome of aging and is considered at least partially reversible. Recent studies, including clinical trials, systematic reviews, and meta‐analyses, consistently demonstrate that frailty is a stronger predictor of clinical outcomes than age alone [3, 14, 36]. Unlike chronological aging, frailty is a potentially reversible condition that can be improved or downgraded with proper geriatric care. Approximately 10% of individuals over the age of 65 and 25%–50% of those over 85 exhibit some degree of frailty, although these rates may vary across different populations and evaluation methods [85].

In this context, comprehensive geriatric assessment (CGA) plays a key role, as it enables the identification of multidimensional health issues that may affect surgical outcomes [14]. Among these, frailty has emerged as a particularly important predictor of adverse postoperative outcomes. Incorporating frailty assessment into the preoperative evaluation process can enhance risk stratification and guide perioperative management strategies to improve outcomes in older surgical patients.

### Frailty models and clinical relevance

Frailty is a clinical syndrome commonly associated with advancing age, characterised by increased vulnerability and reduced resilience to internal and external stressors [36]. It reflects a decline in physiological reserves across multiple organ systems  [42]. In clinical practice, two primary models of frailty are widely recognised: the physical phenotype model  [26] and the deficit accumulation model [59].

Mitnitski et al. define frailty as the cumulative accumulation of health deficits, including symptoms, clinical signs, functional impairments, and laboratory abnormalities, used to quantify an individual′s overall health status and estimate proximity to death  [59]. In contrast, Fried et al. characterise frailty using a physical phenotype, defined by the presence of at least three out of five criteria: low grip strength, self‐reported exhaustion, slow walking speed, low physical activity, and unintentional weight loss [26]. However, frailty is now more widely acknowledged as a multidimensional concept that includes four main domains: physical, cognitive, psychological and social [33].

In perioperative settings, frailty is associated with higher risks of postoperative complications, mortality, extended hospital stays, and long‐term functional decline [84]. The 5‐item modified Frailty Index (mFI‐5) is the most commonly used frailty assessment tool in orthopaedic surgery [36, 84]. Frail individuals are particularly prone to rapid health deterioration following even minor stressors, such as changes in medication or minor infections, as well as major interventions like surgery.

### Advancing age and frailty

Aging is an inevitable and irreversible aspect of human life. However, an individual′s functional capacity is not directly correlated with chronological age [14, 57]. Factors such as healthy nutrition, regular exercise, and positive lifestyle habits can minimise functional decline independently of age [57]. Furthermore, it is important to recognise that the reduction in functional capacity can vary significantly among older adults. With increasing life expectancy and a more active aging population, the expectation of functional longevity has become widely accepted in modern society.

The vascular system is among the most affected by aging and frailty [57]. Moreover, both age and frailty are direct risk factors for impaired vascularisation and reduced tendon elasticity. These changes contribute to an increased risk of bone and ligament‐tendon injuries. The most notable examples of age‐related tendon vascularity and elasticity loss are seen in the Achilles tendon and rotator cuff tendons [57]. Ceker et al. demonstrated that Achilles tendon elastography serves as a highly reliable predictor of frailty, reflecting the age‐related decline in tendon elasticity [6].

Since the seminal work of Brewer in the 1970s, it has been well established that the prevalence of rotator cuff pathology increases with age, affecting up to 70% of individuals by their 70s [5]. Brewer and colleagues further demonstrated that both cellularity and vascularity of the rotator cuff tendons decline significantly after the age of 70, which has important implications for healing and tissue quality. Cadaveric studies have reinforced this understanding, particularly highlighting that osteoporotic changes in the greater tuberosity reduce fixation strength, posing additional challenges in the older population. Nevertheless, leaving these patients untreated is not a viable option [24].

Ducasse and Collin conducted a study comparing clinical outcomes of isolated supraspinatus tendon repair between patients over 70 years of age and a control group under 50, highlighting the impact of age on surgical success [21]. Their findings showed that, despite having similar comorbidities and tear configurations, older patients presented with significantly poorer baseline functional scores prior to surgery. However, when operated on by experienced surgeons, these patients demonstrated greater functional improvement postoperatively. Notably, even though tendon healing was less favourable at the 6‐month follow‐up in the older group, clinical recovery remained satisfactory. This study, which was awarded the 2023 Hawkins Award, has served as a strong source of motivation for shoulder surgeons in treating older patients with rotator cuff tears. Moreover, current evidence suggests that surgical repair in patients over the age of 60 yields superior clinical outcomes when compared to conservative management for rotator cuff tears [20].

### Pain and frailty

A strong correlation exists between chronic pain and the severity of frailty, which is likely to impact patient functionality [90]. Patients with frailty may present with exaggerated preoperative pain or experience greater dissatisfaction in the postoperative period. In a study that provided a novel perspective, Kinnucan et al. evaluated 75 patients aged 65 and older with no complaints in their dominant shoulders [49]. The patients were stratified based on their frailty levels. The study found that as frailty scores increased, both American Shoulder and Elbow Surgeons and Constant–Murley scores decreased, providing evidence that frail patients may not achieve the same levels of improvement after rotator cuff repair, both in clinical practice and research settings. These patients often have lower baseline shoulder scores even before intervention. Accurate identification of frail patients guides the sport surgeon toward more predictable clinical outcomes by aligning treatment strategies with patient expectations. Moorthy et al. investigated the relationship between frailty scores and clinical outcomes in over 300 patients who underwent arthroscopic double‐row rotator cuff repair [62]. They found that frail patients reported significantly worse patient‐reported outcomes scores (PROs) and higher pain scores, identifying the modified frailty index (MFI) as the most reliable predictor.

### Sarcopenia and frailty

Sarcopenia, defined as a progressive loss of skeletal muscle mass and function, is closely correlated with frailty. The Phenotype Model of frailty defines frailty based on five clinical criteria. This model is grounded in the concept of sarcopenia, which refers to the progressive loss of skeletal muscle mass and strength. Sarcopenia involves a decline in contractile protein function, where muscle tissue is progressively replaced by fat and fibrous tissue, leading to a reduction in both the number and size of muscle fibres.

Clinical evidence suggests a strong association between the severity of sarcopenia and shoulder pathologies [38]. For instance, Chung et al. demonstrated that patients with chronic, symptomatic full‐thickness rotator cuff tears had significantly higher sarcopenia indices compared to age‐ and sex‐matched controls  [12]. Moreover, the degree of muscle loss was positively correlated with tear size, indicating that patients with larger tears are more likely to exhibit sarcopenic features.

In surgical populations, sarcopenia alone has been identified as an independent predictor of adverse outcomes. Rectus femoris muscle thickness and activity are inversely related to sarcopenia and are likely to negatively influence outcomes of knee surgeries [63]. Additionally, studies have demonstrated a direct correlation between rectus femoris muscle thickness and frailty, highlighting a potential risk of extension strength loss in frail patients undergoing knee sports surgery [29]. These findings underscore the importance of preoperative evaluation of sarcopenia in patients undergoing knee or shoulder procedures, as it may significantly affect both the choice of treatment strategy and overall prognosis.

### Malnutrition and frailty

Malnutrition and frailty share a bidirectional and reinforcing relationship that significantly affects surgical outcomes, particularly in older adults. Malnutrition accelerates the onset and progression of frailty by promoting sarcopenia, immune dysfunction, and impaired physiological reserve [7, 64]. Conversely, frailty contributes to malnutrition through reduced appetite, functional decline, and social isolation, creating a vicious cycle that compromises postoperative resilience [25, 88]. This interaction is particularly critical in knee sports surgery, where early mobilisation and optimal functional recovery are central to successful outcomes. In frail and/or malnourished sport‐related injured patients, delayed wound healing, increased risk of infections, and poor rehabilitation engagement are frequently observed [9].

Adequate nutrition supports tenocyte metabolism and tendon healing by promoting matrix synthesis and reducing inflammation. Given that tendinopathies account for 30%–50% of sports injuries and are influenced by lifestyle factors such as diet, multifactorial nutritional interventions appear more effective than single‐nutrient approaches [80]. Early identification and management of both frailty and malnutrition, including comprehensive nutritional assessment and targeted interventions, are therefore essential for improving surgical tolerance, minimising complications, and supporting functional recovery in this vulnerable patient population [8, 18].

### Osteopenia and frailty

Osteoporosis and frailty are closely interrelated syndromes that commonly coexist in older adults, both significantly influencing surgical risk and recovery [15]. While advancing age is the most substantial risk factor for osteoporosis, also play key roles in its development [17]. Frailty further contributes to bone loss through mechanisms such as sarcopenia, chronic inflammation, and reduced mobility, creating a cycle of declining musculoskeletal integrity, which is exacerbated by vitamin D deficiency [30, 87] [22]. This condition can lead to tunnel‐related complications in knee surgeries or reduced anchor fixation strength in procedures such as rotator cuff repair or meniscal root repair. Low vitamin D levels and osteopenia may also pose challenges for sports surgeons performing periarticular knee osteotomies, potentially leading to delayed or impaired bone healing. Bone mineral density (BMD) declines with age, particularly in postmenopausal women, due to impaired vitamin D metabolism. According to current literature, this reduction in BMD is a potential independent risk factor for postoperative tendon‐to‐bone healing failure [11]. In frail patients, sports surgeons should maintain a high index of suspicion for reduced BMD, osteopenia, and an increased risk of both intraoperative and postoperative complications.

### Polypharmacy and frailty

Polypharmacy and frailty frequently coexist in older adults, contributing to a cumulative decline in physiological resilience. Frail individuals, due to the burden of multimorbidity, are often exposed to multiple concurrent medications, increasing the risk of drug–drug interactions, adverse effects, falls, cognitive impairment, and hospitalisations [32, 54]. At the same time, inappropriate or excessive pharmacologic treatment may accelerate the onset or progression of frailty, forming a bidirectional and self‐perpetuating cycle. Among commonly used medications, nonsteroidal anti‐inflammatory drugs (NSAIDs) are particularly relevant in orthopaedic and musculoskeletal conditions yet pose significant risks in older adults. Chronic NSAID use has been associated with gastrointestinal bleeding, renal dysfunction, cardiovascular events, and interference with bone healing, all of which are particularly detrimental in frail patients undergoing surgery [1, 86].

### Delirium and frailty

Delirium is an acute neuropsychiatric syndrome marked by a sudden onset of disturbances in attention, awareness, and cognition. It typically presents with a fluctuating course, where symptoms may vary in severity throughout the day. Delirium most commonly emerges within the first 48 h of hospitalisation and is often triggered by underlying medical conditions, surgical procedures, or environmental stressors. Recognition and management of this clinical state are essential, as delirium is associated with increased morbidity, prolonged hospital stays, and higher mortality rates, particularly in frail older adults. Frailty has been shown to increase the risk of delirium in both acute and elective patients. Since the most common type of delirium is the hypoactive type, it is often overlooked. The British Orthopaedic Association Standard for Trauma and Orthopedics guideline for hip fractures recommends the use of the 4AT delirium screening tool, which assesses alertness, orientation, and attention, and has been validated for use with patients over the age of 65 [75]. Triggers for delirium can be factors such as being in pain, taking analgesia, the fracture itself, dehydration, underlying cognitive impairment, acute kidney injury, infection, requiring an anaesthetic, and anaemia. Proper pain control, optimising sleep, minimising tethers, reorienting with clocks, clear communication, early mobilisation, and good nutrition all improves outcomes and reduce the risk of delirium in this patient group.

### Preoperative management of the frail patient

Early identification of frailty allows clinicians to optimise modifiable risk factors before surgery, including nutritional status, sarcopenia, comorbidities, and polypharmacy. This optimisation, often referred to as ‘prehabilitation,’ may include physical therapy, nutritional support, and medical management, all of which have been associated with improved postoperative outcomes in frail individuals. In orthopaedic sport surgery procedures, especially in ligament repair, knee around osteotomy, and rotator cuff repair, recognising and managing frailty preoperatively is crucial to guide surgical decision‐making, set realistic expectations, and reduce adverse outcomes.

The perioperative period is an example of a major physical stressor for older adults living with frailty [83]. The American College of Surgeons and the American Geriatrics Society (AGS) recommend that older surgical patients be assessed for frailty, and its severity should be documented. Frailty assessment before surgery is not just for predicting outcomes it also supports patient‐centred care. According to the National Institute on Aging and the AGS, evaluating frailty helps align patient and caregiver expectations and improves communication about the goals and risks of surgery. This approach is now becoming a standard part of care, as emphasised in the AGS perioperative guidelines for older adults [10, 71]. This document summarises frailty measurement tools in the preoperative period, implication on organs system, and surgical decision making [60]. On the other hand, some other societies of geriatrics and anaesthesiology recommend using the Clinical Frailty Scale (CFS) in the preoperative period for risk assessment [66]. The CFS is a validated tool for identifying frailty without the need for tests in an acute setting. It is developed by a Rockwood et. al, depending on capabilities of activities of daily living and chronic disease control. Frailty assessment is more suitable than ASA grading for preoperative assessment of older adult patients undergoing major orthopaedic surgery and is an effective complement to ASA grading. The most important factor is the identification of the easy‐to‐administer frailty screening tools by the orthopaedic surgeon′s opinion.

Managing major orthopaedic surgery in older patients requires carefully considering perioperative risks, as geriatric patients are often frail, especially with an aging population. To improve outcomes, a thorough evaluation and risk balancing of anaesthesia, surgery, and comorbidities, using a multidisciplinary approach, is essential [61].

### Knee sport surgery and frailty

To date, no studies in the literature have directly examined the impact of frailty on clinical outcomes following knee around osteotomy or cruciate ligament reconstruction [72]. Koltenyuk et al. analysed data from 3797 patients with multiligamentous knee injuries using the NIS database. The authors evaluated age, sex, frailty, and obesity as predictors of hospital length of stay, adverse discharge, and complication rates. Their ROC curve analysis demonstrated that frailty was the most significant predictor [51]. This finding offers new insight for sports medicine surgeons, as ligament healing depends on healthy soft tissue and bone quality. It also reinforces the notion that elderly patients may still be appropriate candidates for procedures such as anterior cruciate ligament reconstruction or knee around osteotomy.

Certain lower extremity sports‐related injuries are directly associated with aging, vascular compromise, and tendon degeneration [28, 47]. Injuries such as patellar and quadriceps tendon ruptures, achilles tendon tears, and meniscal root tears are closely linked to microvascular insufficiency and tissue degeneration – factors known to correlate with frailty and considered potential predictors of it [2, 6]. Moreover, extensor mechanism disruptions, which are more frequently observed in patients with renal or hepatic disease undergoing replacement therapy, represent classic examples of frailty‐associated injuries [68, 79]. In such cases, the surgeon must rely on clinical judgement, considering the patient′s vascularisation status, BMD, and degree of frailty, when deciding between primary repair and reconstruction. Moreover, it should be noted that frail patients undergoing lower extremity surgery require more than twice as much rehabilitation compared to those undergoing upper extremity procedures [48].

Frailty is not limited to individuals aged 65 and older; it can also affect patients over the age of 40, albeit at lower severity [78]. Further research is needed to investigate the correlation between frailty and periarticular knee injuries in this younger, yet still socially and physically active population. For orthogeriatricians, rectus femoris muscle thickness is a key parameter in diagnosing frailty, and this information should alert knee sports surgeons to the potential risk of extensor mechanism weakness and injury in frail individuals [29].

### Shoulder surgery and frailty

Shoulder complaints are highly prevalent among older adults, making the rotator cuff tendons one of the most illustrative models of age‐related chronic degeneration [69]. In degenerated shoulder muscles, fatty infiltration and muscle atrophy are hallmark features that distinguish them from the healthier musculature seen in younger individuals. Advanced age and dyslipidemia have been associated with an increased risk of tendon delamination [44, 52]. These delaminated tears often present as large, complex lesions with poor tendon quality, significant retraction, and prolonged symptom duration, creating substantial surgical challenges [52]. Thickening of the extracellular matrix has been implicated as a key factor in the chronic, age‐related degeneration and breakdown of muscle tissue [69]. In their editorial, Heerspink et al. discussed the importance of identifying rotator cuff tears that are more likely to deteriorate over time and emphasised the value of clinical prediction in guiding the decision not to operate [40].

Fatty infiltration of the infraspinatus muscle, is recognised as important clinical negative prognostic indicators in rotator cuff pathology [40, 44, 53]. In a study by Huang et al., patients with dorsal degenerative disease were stratified into two groups based on frailty scores: Group 1 (FI = 2) and Group 2 (FI = 3–5). The researchers investigated fatty infiltration in the paraspinal muscles and found that frail patients exhibited significantly higher percentages of fatty atrophy and reduced muscle volume, indicative of sarcopenia [43]. The level of frailty shows a strong inverse correlation with fatty atrophy in the paraspinal muscles, which may serve as a potential clinical determinant in the treatment of irreparable rotator cuff tears using lower trapezius and latissimus dorsi tendon transfers. There is currently no definitive evidence in the literature regarding the impact of frailty on shoulder tendon transfers; however, the likely reason for their underuse in older adults is the increasing level of frailty associated with advanced age. In addition, the literature consistently agrees that in patients with proximal humerus fractures and those undergoing reverse shoulder arthroplasty, higher levels of frailty are associated with poorer outcomes and increased complication rates [41, 66].

Additionally, when performing surgery on frail patients, a shoulder surgeon must remain cognisant of potential comorbidities associated with frailty, such as cardiovascular instability or osteopenia. Surgical management should then be guided by the surgeon′s clinical suspicion and intraoperative judgement. For example, the use of additives like adrenaline or tranexamic acid in arthroscopic irrigation fluids may pose cardiovascular risks in frail individuals. Similarly, performing subacromial decompression with a high‐speed burr in a patient with poor bone quality may unexpectedly result in excessive bone loss.

## INTRAOPERATIVE MANAGEMENT OF FRAIL PATIENT PATIENTS

### Anaesthesia management in older adults

Both regional and general anaesthesia are viable options in older patients; however, the anesthesiologist must be familiar with the physiological alterations associated with aging [39]. Regional anaesthesia significantly facilitates shoulder surgery by protecting older patients—considered a high‐risk group—from complications such as urinary retention, bradycardia and hypotension. Advanced age and reduced muscle mass increase sensitivity to anaesthetic agents, and if patient‐specific dose adjustments are not made, there is a high risk of overdose, which may lead to delayed recovery [39]. Ambulatory anaesthesia offers several advantages in older adults, including faster recovery, higher patient satisfaction, reduced healthcare costs, and lower risk of infections and thromboembolic events [39]. Moreover, cognitive recovery is generally superior in ambulatory settings compared to prolonged hospital stays.

Polypharmacy, common in frail patients, can increase anaesthetic depth, making the use of short‐acting agents a more prudent choice [27]. Airway management in frail patients also presents challenges, as these patients may have poor dentition or limited cervical spine mobility. In such cases, a laryngeal mask airway may be preferable to endotracheal intubation, given its association with fewer airway‐related complications.

While the choice between regional and general anaesthesia is often based on the anesthesiologist′s preference and experience, regional anaesthesia may offer several advantages in frail patients, including shorter hospital stays, better postoperative pain control, improved intraoperative hemodynamic stability (particularly critical in procedures like subacromial arthroscopy), higher patient‐reported outcomes and earlier mobilisation [39].

Frail patients are also at increased risk of perioperative hypothermia, which must be actively managed. Importantly, frailty should be recognised as a confounding factor when evaluating poor postoperative outcomes in elderly patients. It can significantly influence anaesthetic planning. Recommendations for optimal management include avoiding potentially inappropriate medications such as benzodiazepines for premedication and monitoring anaesthesia depth to prevent excessive dosing.

### Intraoperative challenges in shoulder surgery in frail patients

In shoulder surgery, intraoperative challenges in frail patients are multifactorial and complex. This section aims to highlight key difficulties that may arise in this population.

Osteopenia significantly reduces anchor pullout strength, particularly in the greater tuberosity, and negatively impacts tendon‐to‐bone healing. Entezari and Lazarus proposed increasing the number of anchors and utilising larger suture anchors to enhance fixation strength [22]. Tingart et al. demonstrated that metallic anchors have significantly higher pullout strength than bioabsorbable anchors [82]. Additionally, inserting anchors perpendicularly (at the so‐called ‘deadman′s angle’ of 45°) can further improve fixation strength [82].

To address poor bone quality caused by osteoporotic resorption or subchondral cystic changes, bone grafting and cement augmentation have been explored. While structural bone grafting does not provide immediate mechanical benefit, materials such as tricalcium phosphate and polymethylmethacrylate have been shown to increase anchor fixation strength significantly [31, 65]. However, the potential for intra‐articular extravasation remains a notable challenge with these materials.

Rotator cuff muscle fatty infiltration, commonly seen in older patients, can impede footprint reduction and tendon mobilisation. In such cases, alternative suture configurations, tendon releases, or mobilisation techniques may be necessary [22, 67]. Furthermore, delamination—often associated with higher levels of fatty infiltration and reduced tendon quality—presents an additional technical obstacle.

### Intraoperative challenges in knee surgery in frail patients

Frailty is known to increase fracture risk through multiple mechanisms, including osteopenia, impaired muscle contraction, and reduced tendon vascularity. These mechanisms, often accompanied by falls, have been linked to various types of fractures [19, 55]. Moreover, aging‐related knee osteoarthritis reduces patients' mobility and exercise capacity, thereby contributing to increased frailty [58]. In essence, total knee arthroplasty (TKA) in frail patients is part of a reciprocal process—osteoarthritis exacerbates frailty, and frailty negatively affects TKA outcomes [89].

Frailty is also relevant not only in arthroplasty but in other conservative and surgical treatments. For instance, when performing an open wedge high tibial osteotomy on a frail patient with suspected osteopenia and potential delayed union, surgeons should be more inclined to use grafts to fill the osteotomy gap. Similarly, during ligament repair procedures, the surgeon must consider the reduced vascularity in tendons of frail individuals, which may predispose them to re‐rupture [74]. Although there are no prospective studies directly evaluating the effect of frailty on clinical outcomes and complication rates following meniscal root repairs or patellar/quadriceps tendon ruptures, this area warrants future investigation. Notably, the higher morbidity and mortality observed in frail patients following patellar fractures already raises clinical concern [76].

Exercise is undoubtedly the most effective strategy for reducing or slowing the progression of frailty [70]. However, postoperative physical therapy in frail patients must be approached with caution [34]. Following knee around tendon repair or reconstruction, early and aggressive rehabilitation may lead to complications such as re‐rupture or fracture in this vulnerable population. Therefore, rehabilitation protocols should be carefully individualised and closely monitored in frail patients to balance functional recovery with surgical protection.

### Postoperative management of the frail patients

Evaluating postoperative recovery in orthopaedic patients should extend beyond mortality rates. For patients and their families, mobility and particularly ambulation are often the primary concern [70]. Therefore, a comprehensive understanding of the risk factors contributing to postoperative ambulation impairment is essential for optimising functional outcomes and guiding patient‐centred care [23]. While previous studies primarily focused on mortality rates, there is now increased attention on the recovery of postoperative ambulation. This shift is driven by the recognition that postoperative ambulation is of significant concern to patients and their families.

**FIGURE 2:**
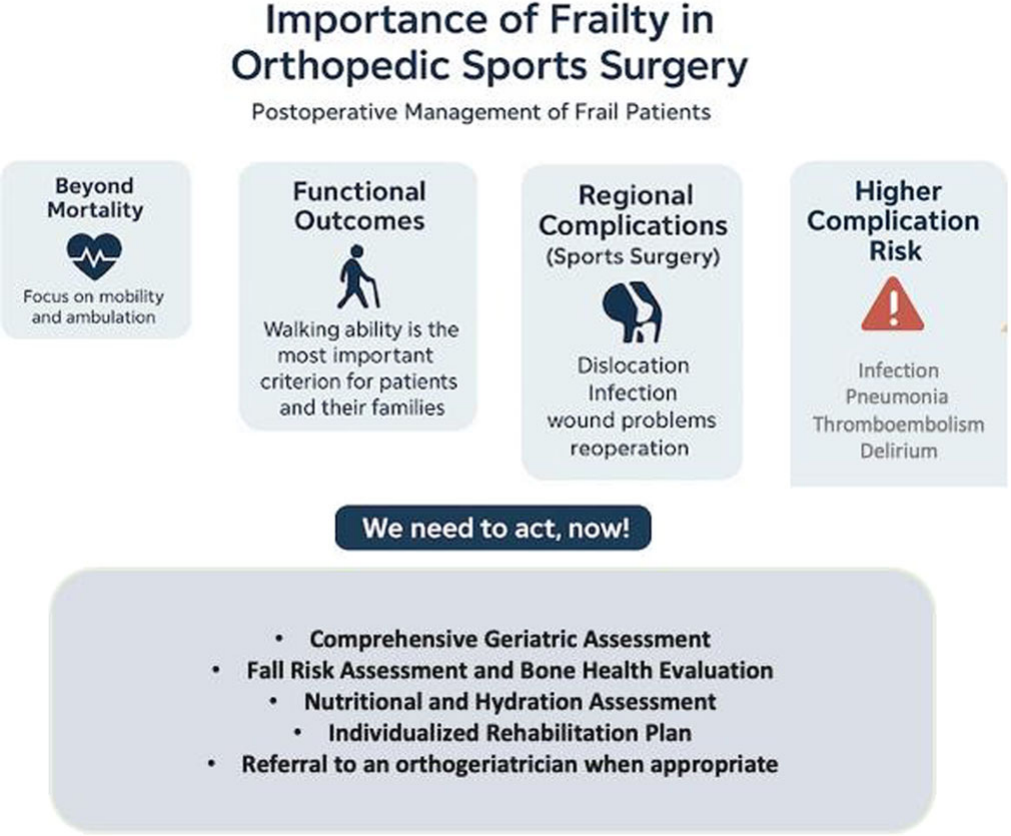
This infographic summarises postoperative challenges and management of frail patients.

Hall et al. examined how preoperative frailty screening affects postoperative outcomes. After implementing frailty screening and developing a multidisciplinary care plan based on geriatric principles—including palliative care consultations for high‐risk patients—the 30, 180, and 365 days significantly declined. This indicates that recognising and addressing frailty before surgery can enhance surgical results [37]. Postural analysis of patients identified as frail may facilitate the development of targeted rehabilitation programmes aimed at improving posture and independent ambulation, thereby potentially enhancing the effectiveness of region‐specific postoperative rehabilitation, whether for the knee, shoulder, hip or ankle (Figure 2).

### Postoperative complications and frailty

Frail patients undergoing orthopaedic surgery face higher risks of complications, more extended hospital stays, and readmission [36]. Increased frailty links to more Clavien–Dindo Class IV complications and hospital‐acquired conditions like infections, pneumonia, thromboembolism, and urinary tract infections across different procedures. Additionally, other postoperative complications, like myocardial infarction or pulmonary embolism, may initially manifest as delirium in older adults [56].

In addition to systemic complications, frailty may contribute to regional complications in knee and shoulder sports surgery patients. These include wound infections triggered by frailty and malnutrition, prolonged hospital stays [51], or failed repairs [62, 91]. Therefore, patients should be closely monitored and evaluated at more frequent follow‐up intervals.

### Postoperative rehabilitation and social frailty

The postoperative rehabilitation of older adults, particularly those living with frailty, demands an individualised approach that goes beyond standard physical recovery protocols. The European Geriatric Medicine Society has recently introduced a consensus document emphasising the importance of CGA as an integral component of rehabilitation planning [35]. This structured assessment forms the foundation for a meaningful shared decision‐making process, which is particularly crucial in the context of frailty, where treatment goals may diverge significantly from traditional curative models. Furthermore, adherence to the rehabilitation plan and ongoing monitoring of progress must be actively supported throughout the recovery trajectory. These elements are essential to promoting a sustainable, participatory rehabilitation process that respects the fluctuating functional reserves and vulnerabilities associated with frailty. Ultimately, integrating CGA into postoperative care facilitates the development of person‐centred rehabilitation strategies that not only enhance recovery outcomes but also uphold the dignity, autonomy, and preferences of older adults during one of their most critical phases of care.

In this context, a new concept is encountered, known as social frailty, which reflects a patient′s ability to adhere to postoperative treatment protocols and rehabilitation [4]. Frail patients are more likely to be lost to follow‐up after surgery, which may result from poor adherence to rehabilitation [62]. Postoperative non‐compliance may be attributed to several factors, including systemic complications, lack of independent access to rehabilitation centres, or mood‐related motivational issues. Surgeons should not apply the same rehabilitation programme to patients across all age groups and frailty levels. Even if similar protocols are prescribed, the level of adherence in patients under 50 cannot be expected to match that of older individuals [50].

### Patient‐reported outcomes and frailty

Frailty is not only associated with increased mortality but also elevates the risk of postoperative complications such as dislocation, infection, wound problems, and reoperation [46, 67]. Although frailty negatively impacts PROs in various orthopaedic procedures, further comprehensive research is needed in this area [16, 36].

In a study by Moorty et al., the relationship between frailty scores and clinical outcomes was examined in over 300 patients who underwent arthroscopic double‐row rotator cuff repair. The study demonstrated that frail patients reported poorer clinical outcomes and higher pain levels, identifying the MFI as the most predictive classification system [62].

It is important to note that the outcome measures used in shoulder and elbow sports surgery not only assess limb‐specific function but also reflect the patient′s overall well‐being and satisfaction. Given that elderly patients typically present with lower baseline scores, it would be inappropriate to evaluate PROs of frail and non‐frail patients within the same category.

### We need to act, now!

The british orthopaedic association has proposed a management framework for trauma patients [75]. Although this pathway may not be directly applicable to sports surgery, it can be adapted by surgeons based on their own clinical experience. While frailty is not strictly correlated with chronological age, orthopaedic sports surgeons should be familiar with the concept and remain alert to the systemic and local complications associated with frailty.

In such cases, a referral for CGA by an orthogeriatrician may be appropriate. Selected patients should undergo multifactorial falls risk assessment and bone health evaluation, followed by suitable interventions and referrals. Nutritional assessment should also be considered; unnecessary preoperative fasting should be avoided unless surgery is imminent, and patients should be encouraged to maintain hydration.

Finally, social frailty significantly influences postoperative congruence and rehabilitation engagement. Importantly, frailty is both modifiable and manageable, emphasising the need for individualised care planning in this vulnerable population.

## CONFLICT OF INTEREST STATEMENT

The authors declare no conflicts of interest.

## ETHICS STATEMENT

In accordance with federal regulations, this research study has been determined to be exempt from Institutional Review Board (IRB) oversight. The exemption status is based on the nature of the research, which involves no risk to participants and does not involve sensitive or personally identifiable information.
